# Transcriptome Analysis of the Regulatory Mechanisms of Holly (*Ilex dabieshanensis*) under Salt Stress Conditions

**DOI:** 10.3390/plants13121638

**Published:** 2024-06-13

**Authors:** Hong Chen, Huihui Li, Xinran Chong, Ting Zhou, Xiaoqing Lu, Xiaolong Wang, Bingsong Zheng

**Affiliations:** 1 Jiangsu Key Laboratory for the Research and Utilization of Plant Resources, Institute of Botany, Jiangsu Province and Chinese Academy of Sciences, Nanjing Botanical Garden Mem. Sun Yat-Sen, Nanjing 210014, China; 2 Zhejiang Provincial Key Laboratory of Forest Aromatic Plants-Based Healthcare Functions, Zhejiang A & F University, Hangzhou 311300, China; 3 Fuyang Academy of Agricultural Sciences, Fuyang 236065, China

**Keywords:** salt stress, transcriptome, DEGs, plant hormone signal transduction, antioxidant, osmoregulation

## Abstract

The holly *Ilex dabieshanensis* K. Yao & M. B. Deng, a tree endemic to the Dabieshan Mountains region in China, is a commonly used landscaping plant. Like other crops, its growth is affected by salt stress. The molecular mechanism underlying salt tolerance in holly is still unclear. In this study, we used NaCl treatment and RNA sequencing (RNA-seq) at different times to identify the salt stress response genes of holly. A total of 4775 differentially expressed genes (DEGs) were identified. Kyoto Encyclopedia of Genes and Genomes (KEGG) analysis of the DEGs obtained at different salt treatment times (3, 6, 9, 12, and 24 h), as compared to control (ck, 0 h), showed that plant hormone signal transduction and carotenoid biosynthesis were highly enriched. The mechanism by which holly responds to salt stress involves many plant hormones, among which the accumulation of abscisic acid (ABA) and its signal transduction may play an important role. In addition, ion homeostasis, osmotic metabolism, accumulation of antioxidant enzymes and nonenzymatic antioxidant compounds, and transcription factors jointly regulate the physiological balance in holly, providing important guarantees for its growth and development under conditions of salt stress. These results lay the foundation for studying the molecular mechanisms of salt tolerance in holly and for the selection of salt-tolerant varieties.

## 1. Introduction

Soil salinity stands out as a significant abiotic stressor that impacts plant growth. Salt stress can lead to an imbalance of ions inside and outside plant cells, leading to alterations in osmosis and thereby affecting plant growth and development. The plant salt stress response is a complex process involving multiple systems, including signal transduction, transcription factor regulation, and gene expression regulation [1,2,3].

There is increasing evidence for the important role of the salt overly sensitive (SOS) signal pathway in plant salinity tolerance [4,5]. Under conditions of high salinity, Na^+^ will enter plant cells, leading to an increase in intracellular Na^+^ concentration. High Na^+^ concentrations inside or outside the cell can cause an increase in intracellular Ca^2+^ concentration, triggering Ca^2+^ signaling [1]. The EF-hand type Ca^2+^-binding protein SOS3 acts as a sensor and binds with Ca^2+^ to activate the serine/threonine protein kinase SOS2. The SOS3-SOS2 complex formed can mediate the activity of SOS1 protein, the plasma membrane Na^+^/H^+^ reverse transporter protein, thereby releasing Na^+^ from the cytoplasm [4]. In addition, SOS2 can mediate the activities of other transporter proteins, such as NHX and H-ATPase. Various SOS2-like proteins (PKSs/CIPKs) can interact with one or more of various SOS3-like Ca^2+^-binding proteins (SCaBPs/CBL) [6]. Under conditions of salt stress, chaperone J3 inhibits the activity of PKS5 to relieve the effects of PKS5 on plasma membrane H^+^-ATPase inhibition [7]. The SOS pathway mediates ion homeostasis, thereby enhancing plant tolerance to Na^+^ stress [8]. In addition to ion levels, some plant metabolites, including polyols, glycine betaine, sugars, and proline, are used as osmotic regulators to mediate the osmotic balance of plant cells under conditions of salt stress [9].

Salt stress causes the accumulation of reactive oxygen species (ROS) in plants. At the same time, salt stress induces enzyme and nonenzyme scavengers to decrease ROS stress, such as SOD, CAT, APX, ascorbic acid, carotenoids, and flavonoids [10]. ROS can act as second messengers and participate in salt stress signal transduction pathways [10]. H_2_O_2_ can activate mitogen-activated protein kinases (MAPKs). The mitogen-activated protein kinase (MAPKKK-MAPKK-MAKK) cascade, as an important signal transduction pathway, is involved in regulating ion homeostasis, ROS homeostasis, and plant growth [11]. Under conditions of salt stress, AtMPK6 interacts with PLD1-derived phosphatidic acid (PA) to phosphorylate and activate SOS1 and thus mediate ion homeostasis [12]. The MAPK cascade reaction is also an important detoxification signaling pathway that participates in antioxidant defense reactions and regulates ROS homeostasis in response to salt/oxidative stress [11]. In this process, there is also some crosstalk with plant hormone signaling pathways. Under conditions of salt stress, the calcium-dependent pathway activates abscisic acid (ABA) biosynthesis-related genes, and the increase in ABA signal can upregulate the salt tolerance-related MAPK family. Elevated ABA levels regulate osmosis by closing stomata and inducing the accumulation of large amounts of proteins and osmotic protectants [3]. The MEKK1-MKK1/MKK2-MPK4 cascade plays a crucial role in stress signaling initiated by ROS and salicylic acid (SA). SA can prevent salt-induced K^+^ loss and reduce the accumulation of Na^+^ in the aboveground parts of the plant, thereby improving the salinity tolerance of *Arabidopsis* [2]. MKK9 mediates ethylene biosynthesis by activating MPK3/MPK6 and participates in salt stress responses in *Arabidopsis* [13]. Transcription factors (TFs) can act as key regulators of various signaling pathways to control the transcription of target genes further downstream [14]. The bZIP, WRKY, AP2, NAC, C2H2 zinc finger genes, and dehydration response element binding (DREB) family include large numbers of molecules involved in responses to stress [2]. The overexpression of heat shock factor HSFA4A enhances the tolerance of *Arabidopsis* to salt and oxidants, and HSFA4A is a substrate of MPK3/MPK6 [15]. Under conditions of salt stress, salt-responsive ERF1 (SERF1) can bind to the promoters of MAPK kinase kinase kinase 6 (MAP3K6), MAPK5, DREB2A, and zinc finger protein 179 (ZFP179), regulating ROS-dependent signaling [9].

The holly *Ilex dabieshanensis* (*I. dabieshanensis*), a tree endemic to the Dabieshan Mountains region in China, is an important landscaping plant that has good ornamental and ecological adaptability. Studying the mechanism of salt stress response will be useful for improving the salt tolerance of holly and promoting its cultivation and application in areas of high salinity. At present, there have been few reports on the salt tolerance of holly. Sucrose has been shown to improve the tolerance of *Ilex aquifolium* L. (English holly) to NaCl by promoting the accumulation of carotenoids, lutein, and chlorophyll [16]. Four types of urban trees were subjected to salt stress treatment. Based on the degree of damage to the leaf photosynthetic system (Fv/Fm value), English holly showed poorer salt tolerance than *Quercus ilex* (Pirnal oak) [17]. However, no further data are currently available indicating the specific molecular mechanism underlying the regulation of holly under conditions of salt stress. The complete genome sequence of *Ilex polyneura* has been obtained, laying an important foundation for molecular-level research in the genus *Ilex* [18]. Previously, we treated *I. dabieshanensis* seedlings with a range of NaCl concentrations (0, 100, and 200 mM) for 4 weeks and found that significant phenotypic changes were observed in holly seedlings treated with 200 mM NaCl (Figure S1), which was consistent with the salt concentration used in other reports [19,20]. Therefore, in this study, we analyzed the variation in transcript levels of *I. dabieshanensis* under different salt stress treatment times (0, 3, 6, 9, 12, and 24 h) [21], providing new insights into the response mechanism of *I. dabieshanensis* to salt stress.

## 2. Results

### 2.1. Differential Gene Expression Analysis

**Figure 1:**
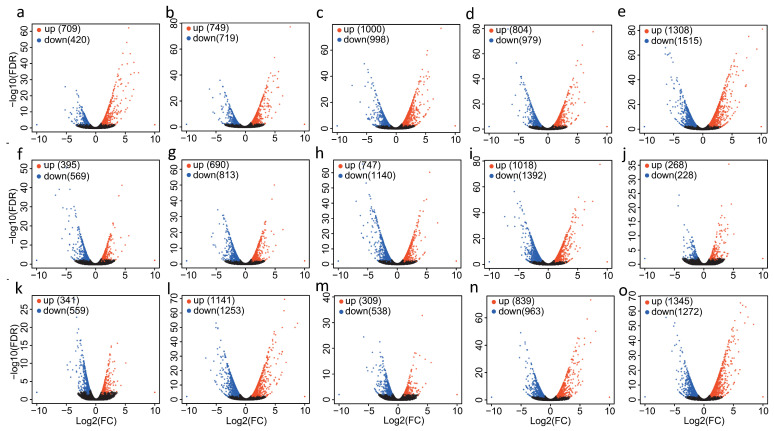
Volcano-plots of salt-induced DEGs. (**a**) 3 h vs. ck (0 h); (**b**) 6 h vs. ck; (**c**) 9 h vs. ck; (**d**) 12 h vs. ck; (**e**) 24 h vs. ck; (**f**) 6 h vs. 3 h; (**g**) 9 h vs. 3 h; (**h**) 12 h vs. 3 h; (**i**) 24 h vs. 3 h; (**j**) 9 h vs. 6 h; (**k**) 12 h vs. 6 h; (**l**) 24 h vs. 6 h; (**m**) 12 h vs. 9 h; (**n**) 24 h vs. 9 h; (**o**) 24 h vs. 12 h. The significantly up-regulated genes are represented by red dots, the significantly down-regulated genes are represented by blue dots, and the genes that do not show significant differential expression are represented by black dots.

To study the gene expression of holly under salt stress, variations in transcription were analyzed under different salt treatment times. The results showed that salt stress induced a total of 4775 differentially expressed genes (DEGs) and that the number of DEGs was related to the period of salt stress (Figure 1, Table S1). Compared with normal growth conditions, longer periods of salt stress resulted in more DEGs (Figure 1a–e). For example, 3 h vs. ck showed 709 upregulated genes and 420 downregulated genes, while 24 h vs. ck showed 1308 upregulated genes and 1515 downregulated genes (Figure 1a,e). In addition, there were significant differences in the expression of some genes between different stress treatments. As the gap between stress times increased, the number of DEGs also showed an upward trend (Figure 1f–o). For example, 6 h vs. 3 h had 749 upregulated genes and 719 downregulated genes, while 24 h vs. 3 h had 1018 upregulated genes and 1392 downregulated genes (Figure 1f,i).

### 2.2. GO Enrichment Analysis of DEGs

**Figure 2:**
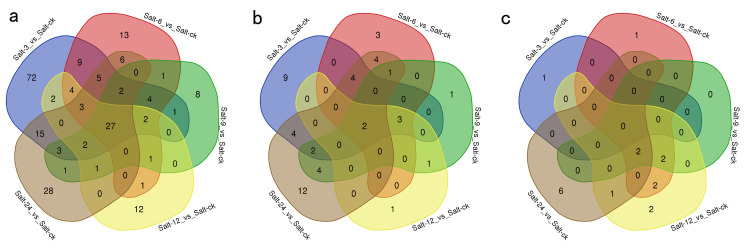
Venn diagram of overlapping DEGs GO enriched terms in holly under salt stress for 3, 6, 9, 12, and 24 h. (**a**) Biological process; (**b**) cellular component; (**c**) molecular function.

Gene ontology (GO) enrichment analyses of DEGs influenced by various salt treatments revealed a substantial enrichment of DEGs associated with biological processes, molecular functions, and cellular components. In this study, we used an adjusted *p*-value of <0.05 as the criterion for determining significant enrichment in our study. This implies that only gene pathways or sets showing adjusted *p*-values below 0.05 were regarded as significantly enriched. A total of 223 DEGs were related to biological processes (Figure 2a). Among them, 27 terms were enriched in different stress times, including some response processes (response to abiotic stimulus, response to oxygen-containing compound, response to water deprivation, and response to hormone), signal transduction, and circadian rhythm (Figure 2a, Table S2). The DEGs of Salt-3 h were significantly enriched in 72 unique terms, mostly involved in regulatory processes, including regulation of response to stimulus, cellular metabolic process regulation, biosynthetic process regulation, regulation of signal transduction, etc. (Figure 2a, Table S2). In addition, 28 unique terms were enriched during prolonged salt stress (Salt-24 h), including response to salt stress, the oxidation-reduction process, ion transport, carbohydrate metabolic process, etc. (Figure 2a, Table S2). There were 51 GO terms related to molecular function (Figure 2b). Two GO terms were significantly enriched in DEGs under different stress treatments, i.e., DNA-binding transcription factor activity and transcription regulator activity (Figure 2b, Table S3). Short-term salt stress-induced DEGs were specifically enriched in nine GO terms, mainly involving sequence-specific DNA binding, carbon-nitrogen lyase activity, acid phosphatase activity, polygalacturonate 4-alpha-galacturonosyltransferase activity, and phosphoric ester hydrolase activity (Figure 2b, Table S3). The DEGs induced by long-term salt stress were specifically enriched in 12 GO terms, mainly involving transporter activity, phosphatase inhibitor activity, oxidoreductase activity, and transaminase activity (Figure 2b, Table S3). There were only 17 GO terms for cell components, including the plasma membrane, cell wall, thylakoid, apoplast, etc. (Figure 2c, Table S4).

### 2.3. KEGG Enrichment Analysis of DEGs

**Figure 3:**
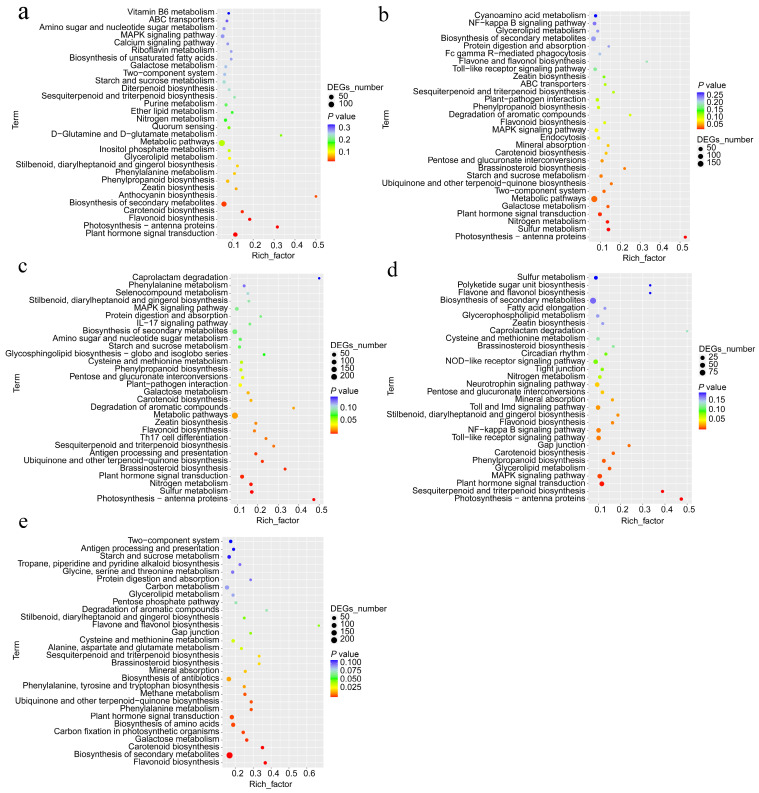
The top 30 KEGG terms of DEGs under salt stress for 3, 6, 9, 12, and 24 h. (**a**) 3 h vs. ck (0 h); (**b**) 6 h vs. ck; (**c**) 9 h vs. ck; (**d**) 12 h vs. ck; (**e**) 24 h vs. ck. The *Y*-axis represents the KEGG pathways, and the *X*-axis represents the Rich factor. The size of the dot indicates the number of differentially expressed genes in the pathway, and the color of the dot corresponds to a different Q value.

To gain deeper insights into the roles of genes associated with the response to salt stress in holly, we examined the top 30 terms of KEGG enrichment triggered by various salt stress treatments. The findings showed that these KEGG terms primarily encompassed metabolism of carbohydrates, synthesis of additional secondary compounds, metabolism of energy, metabolism of lipids, metabolism of amino acids, metabolism of vitamins and cofactors, metabolism of polyketides and terpenoids, and transduction of signals, etc. (Figure 3, Table S5). The five KEGG terms with the highest frequency of occurrence, appearing with all stress treatments, were flavonoid biosynthesis, biosynthesis of secondary metabolites, plant hormone signal transduction, carotenoid biosynthesis, and sesquiterpenoid and triterpenoid biosynthesis (Figure 3, Table S5). DEGs induced by different salt stresses were significantly enriched (*p* < 0.05) in plant hormone signal transduction and carotenoid biosynthesis (Figure 3). DEGs induced at 3, 6, 9, and 12 h were coenriched in five terms: zeatin biosynthesis, MAPK signaling pathway, nitrogen metabolism, photosynthesis-antenna proteins, and phenylpropanoid biosynthesis (Table S5). Photosynthesis-antenna proteins were significantly enriched in all stages of salt stress except 24 h (Figure 3). In addition, terms that could be coenriched by four salt stress treatments include starch and sucrose metabolism, galactose metabolism, glycerolipid metabolism, stilbenoid, diarylheptanoid, and gingerol biosynthesis, and brassinosteroid biosynthesis (Table S5). The calcium signaling pathway was only enriched in DEGs induced by Salt-3 h (Figure 3).

### 2.4. Signal Transduction-Related Genes Differentially Expressed in Response to Salt Stress

#### 2.4.1. Differential Expression of Calcium Signaling Pathway-Related Genes under Conditions of Salt Stress

**Figure 4:**
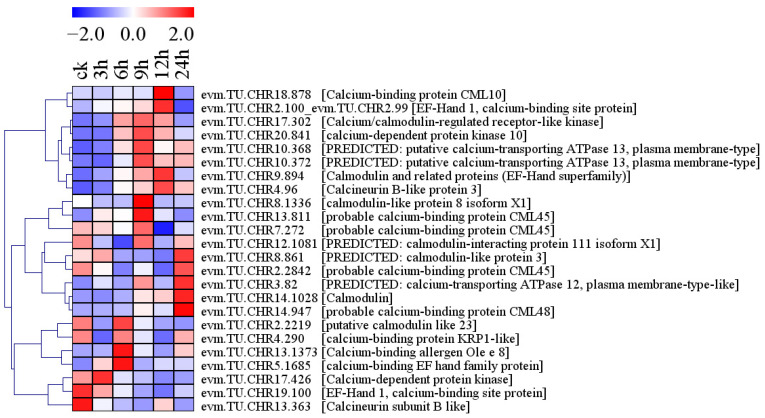
Ca signaling pathway-related genes were differentially expressed in response to salt stress in holly.

Under conditions of salt stress, 24 genes involved in the calcium signaling pathway were differentially expressed in holly, including 12 calcium-binding proteins (CMLs), three calmodulin-like proteins (CALMLs), one calcium/calmodulin-regulated receptor-like kinase (CRCK), one calmodulin-binding protein (CBP), three calcium-transporting ATPases (ACAs), two calcium-dependent protein kinases (CDPKs), and two calcineurin B-like proteins (CBLs) (Figure 4, Table S6). Eleven genes were upregulated by salt stress, including five CMLs, three ACAs, one CRCK, one CBP, and one CBL (Figure 4, Table S6). The level of evm.TU.CHR20.841 expression was upregulated by 3.37-fold after 9 h of salt stress (Table S6). Two genes were downregulated by salt stress, including one CML and one CBL (Figure 4, Table S6). The levels of CML10, CML, and CDPK expression first increased and then decreased with the prolongation of stress time (Figure 4). In contrast, the levels of CALML3 and CML45 expression first decreased and then increased with prolonged stress (Figure 4).

#### 2.4.2. Differential Expression of Protein Kinase Genes under Conditions of Salt Stress

**Figure 5:**
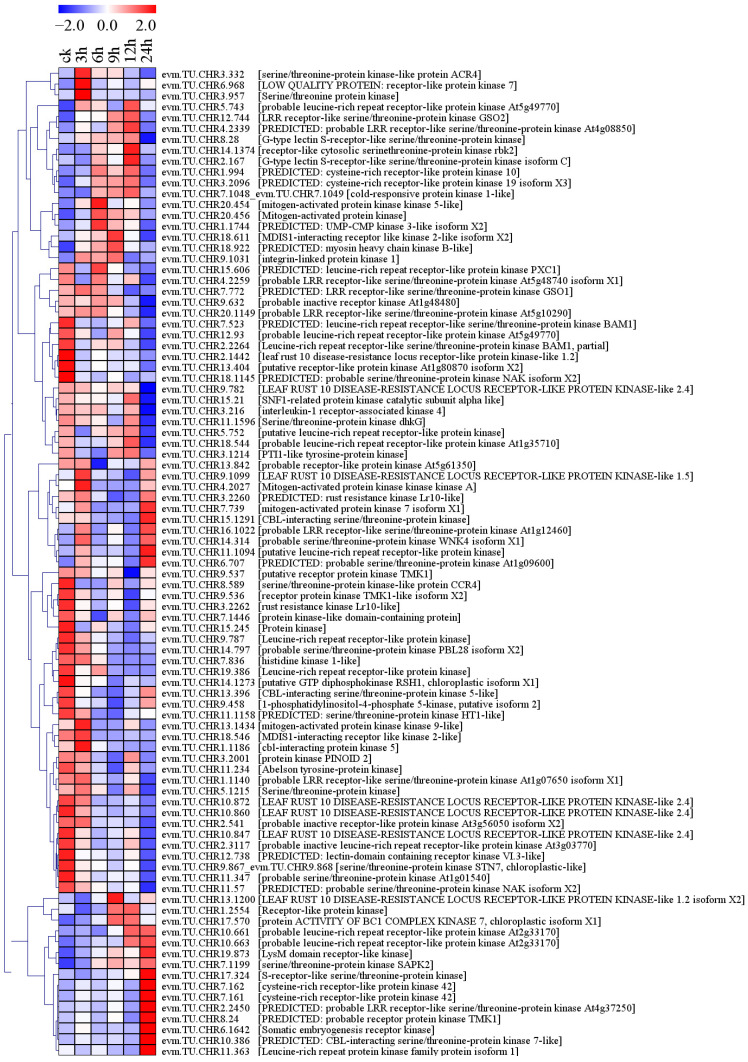
PKs were differentially expressed in response to salt stress in holly.

Protein kinases (PKs) play important roles in the perception and transduction of stress signals. In this study, 91 PK genes were differentially expressed under conditions of salt stress, 34.07% (31/91) of which were members of the serine/threonine protein kinase (STPK) family (Figure 5). These PKs included 53 receptor-like kinases (RLKs), five MAPK cascade proteins, four CBL-interacting protein kinases (CIPKs), one sucrose nonfermenting1 (SNF1)-related protein kinase (SnRK) catalytic subunit alpha-like, and 28 other PKs (Figure 5, Table S7). A total of 18.87% (10/53) of RLKs were upregulated. Among them, evm.TU.CHR10.663 showed almost no expression under normal conditions, and its expression gradually increased with the prolongation of stress time, reaching a FPKM (fragments per kilobase of transcript per million fragments mapped) value of 2.60 after 24 h of salt stress (Table S7). In contrast, 33.96% (18/53) of RLKs were downregulated. The FPKM value of evm.TU.CHR10.872 under normal conditions was 2.09, and it showed almost no expression after 6 h of salt stress (Table S7). According to the annotation results, the five MAPK cascade proteins included two MAPKs, two mitogen-activated protein kinase kinases (MAPKKs), and one mitogen-activated protein kinase kinase kinase (MAPKKK) (Figure 5, Table S7). The MAPK gene evm.TU.CHR20.456 was upregulated under salt stress conditions. The MAPKKK gene evm.TU.CHR4.2027 and the MAPKK gene evm.TU.CHR13.1434 were both initially upregulated and then downregulated by salt stress. The CIPK gene evm.TU.CHR10.386 was upregulated by salt stress (except at 12 h) and showed 8.92-fold upregulation at 24 h compared with 0 h (Figure 5, Table S7).

#### 2.4.3. Differential Expression of Genes Involved in Plant Hormone Signal Transduction under Conditions of Salt Stress

**Figure 6:**
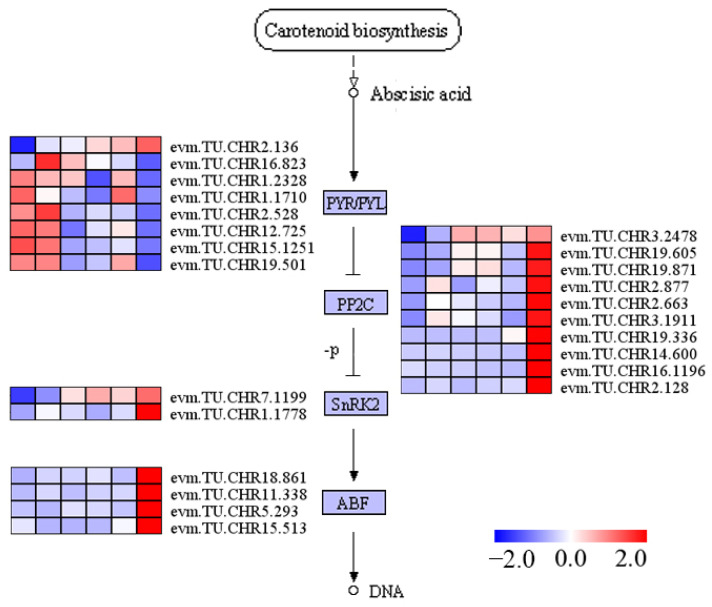
Genes related to the signaling pathway of ABA showed differential expression upon exposure to salt stress in holly. The boxes from left to right depict the conditions of ck, Salt-3 h, Salt-6 h, Salt-9 h, Salt-12 h, and Salt-24 h.

The results of the analysis of plant hormone signal transduction (ko04075) showed that salt stress induced the differential expression of 109 genes in holly, including eight hormones: ABA, auxin, gibberellin, jasmonic acid, ethylene, cytokinin, brassinolide, and SA (Table S8). The ABA signaling pathway is the most widely studied pathway under conditions of salt stress. Twenty-four ABA signaling-related genes were differentially expressed under conditions of salt stress, including PYR/PYL (*n* = 8), PP2C (*n* = 10), SnRK2 (*n* = 2), and ABF (*n* = 4) (Figure 6). More than half of the PYR/PYL genes were downregulated by salt stress, while PP2C, SnRK2, and ABF were almost all upregulated by salt stress (Figure 6). One ABF (evm.TU.CHR18.861) and one PP2C (evm.TU.CHR19.605) were upregulated by more than 13-fold after 24 h of salt stress (Table S8).

### 2.5. Differential Expression of TFs in Holly under Conditions of Salt Stress

**Figure 7:**
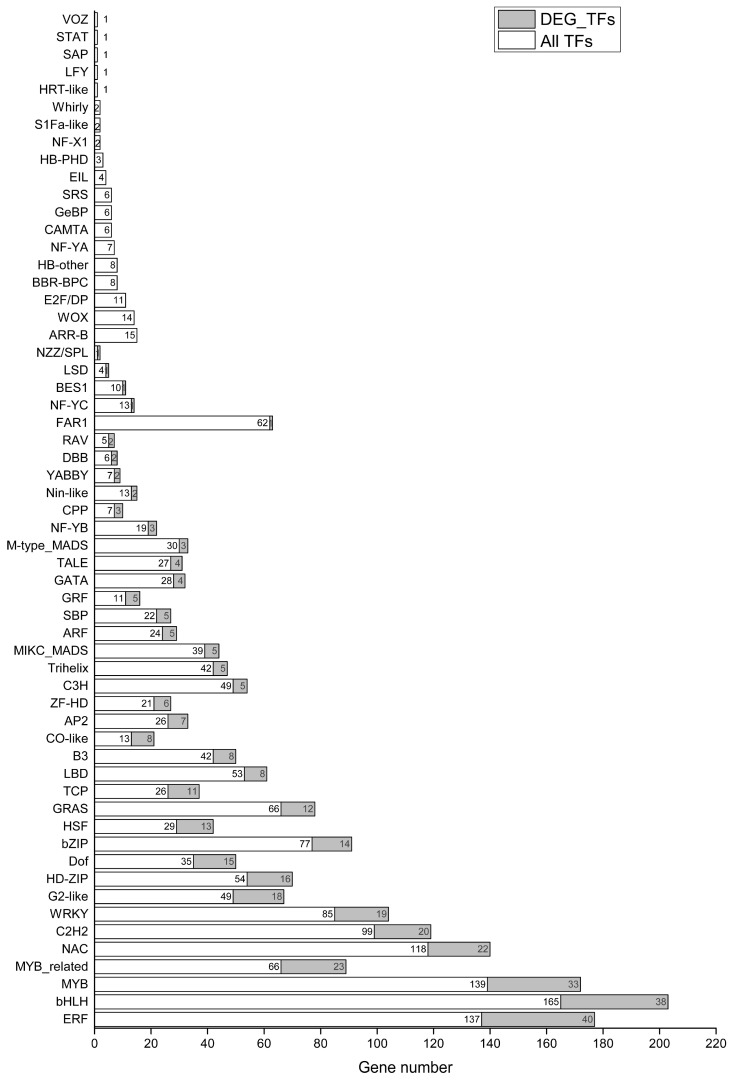
Number of transcription factors in different families differentially expressed under salinity stress in holly. DEG_TFs represent transcription factors in differentially expressed genes, while all TFs represent transcription factors in transcriptome data.

Through RNA-seq, a total of 1818 TFs were found in holly, which were divided into 58 families (Figure 7). Among them, 391 differentially expressed TFs induced by salt stress involved 39 families (Figure 7). Approximately 75% (294/391) of differentially expressed TFs were distributed in ERF (*n* = 40, 10.23%), bHLH (*n* = 38, 9.72%), MYB (*n* = 33, 8.44%), MYB-related (*n* = 23, 5.88%), NAC (*n* = 22, 5.63%), C2H2 (*n* = 20, 5.12%), WRKY (*n* = 19, 4.86%), G2-like (*n* = 18, 4.60%), HD-ZIP (*n* = 16, 4.09%), Dof (*n* = 15, 3.84%), bZIP (*n* = 14, 3.58%), HSF (*n* = 13, 3.32%), GRAS (*n* = 12, 3.07%), and TCP (*n* = 11, 2.81%) families (Figure 7). About half (184/391) of the TFs were upregulated by salt stress, while 30.43% (119/391) of the TFs were downregulated by salt stress (Table S9). Approximately half of the ERFs (21/40) were upregulated, and the FPKM values of three ERFs (evm.TU.CHR17.859, evm.TU.CHR16.1194, and evm.TU.CHR4.443) were <1 under normal conditions but >1 at 3, 6, 9, 12, and 24 h. In particular, evm.TU.CHR16.1194 was significantly upregulated by salt stress (Table S9). Similarly, one MYB gene (evm.TU.CHR2.623) showed significantly upregulated expression at 3, 6, 9, 12, and 24 h. (Table S9). Thirteen genes were upregulated and 10 genes were downregulated under salinity stress among 38 bHLHs (Table S9). Ten MYB-related genes were upregulated by salt stress. The expression level of MYBS3-like (evm.TU.CHR16.931) increased by 4.21-fold under conditions of salt stress compared to control (Table S9). The putative WRKY1b transcription factor evm.TU.CHR1.1507 was downregulated by salt stress for 3, 6, 9, 12, and 24 h and showed almost no expression at 6, 9, and 24 h (Table S9). Under conditions of salt stress, nine HD-ZIPs were upregulated and three HD-ZIPs were downregulated. Among them, the homeobox leucine zipper protein ATHB-13-like isoform X2 (evm.TU.CHR2.2871) was continuously upregulated, reaching 5.60-fold higher than control after 24 h of salt stress. ATHB-12-like (evm.TU.CHR5.2061) was also upregulated 5.93-fold by salt stress for 24 h, at which time the FPKM reached 189.57 (Table S9). Of 14 differentially expressed bZIPs, 10 genes were upregulated by salt stress, including 3 ABSCISIC ACID-INSENSIVE 5-like genes (evm.TU.CHR5.293, evm.TU.CHR18.861, and evm.TU.CHR11.338). In particular, evm.TU.CHR18.861 was upregulated by 1.43-, 1.34-, 1.96-, 0.61-, and 13.21-fold at 3, 6, 9, 12, and 24 h, respectively. Almost all of the 13 differentially expressed HSFs were upregulated by salt stress (Table S9).

### 2.6. Differential Expression of Osmoregulation-Related Genes under Conditions of Salt Stress

#### 2.6.1. Salt Stress Induced the Expression of DEGs Related to Carbohydrate Metabolism

Under conditions of salt stress, 118 genes related to carbohydrate metabolism showed differential expression (Table S10). Thirty-five genes, including three types of kinases (6-phosphofractokinase 3-like protein, inositol 3-kinase, and pyruvate kinase), were upregulated. Inositol 3-kinase (evm.TU.CHR4.2011) was upregulated by 8.02-fold after 24 h of salt stress. Some other genes were upregulated by salt stress, including those encoding galactinol synthase 1-like, probable galactinol-sucrose galactosyltransferase 2 isoform X1, UDP-glucuronic acid decarboxylase 6, UDP-glucose 6-dehydrogenase family protein 1, alpha-1,4 glucan phosphorylase L-1 isozyme, and alpha-1,4-glucan-protein synthase (Table S10). Forty-six genes were downregulated, including alpha-l-arabinofuranosidase, beta-hexosaminidase, fructokinase-2, fructose-1,6-bisphosphatase class 1/Sedoheputulose-1,7-bisphosphatase, glycosyl transferase, phosphatidylinositol 4-phosphate 5-kinase, bifunctional UDP-glucose 4-epimerase, UDP-glucuronate 5-epimerase, UDP-glucuronate 4-epimerase, and UDP-xylose 4-epimerase 1 isoform X2. Under normal conditions, the FPKM of alpha-l-arabinofuranosidase was 31.23, which decreased significantly to 0.37 after 24 h of salt stress (Table S10).

#### 2.6.2. Expression of DEGs Related to Amino Acid Metabolism Induced by Salt Stress

**Figure 8:**
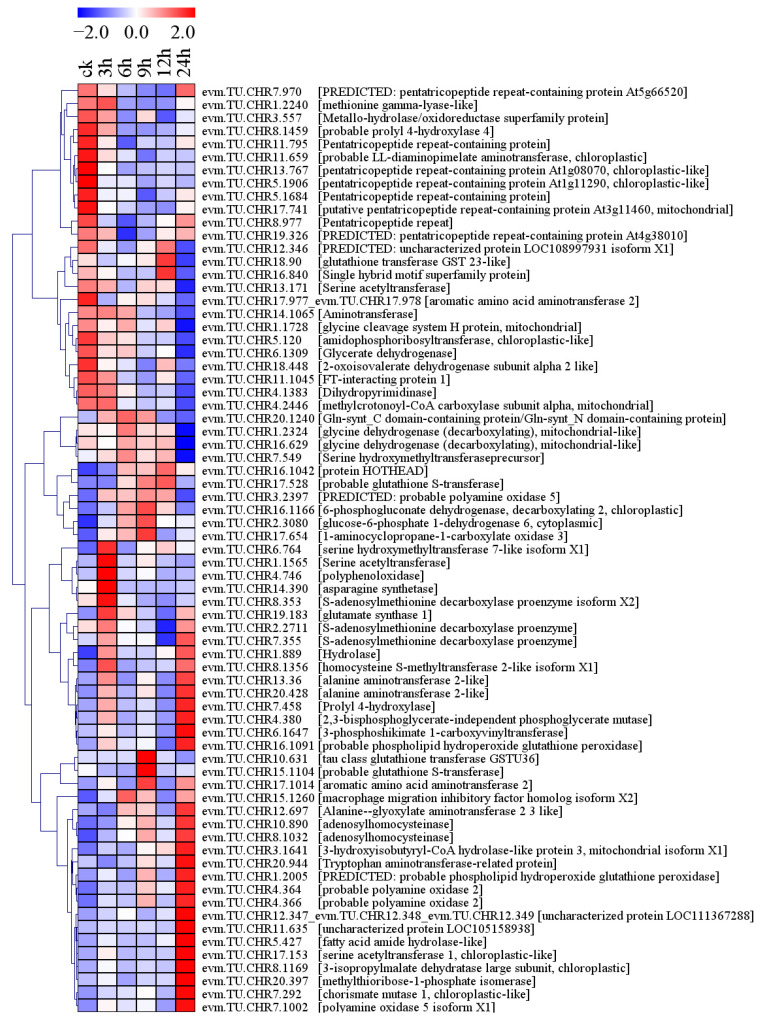
Amino acid metabolism-related genes were differentially expressed in response to salt stress in holly.

Seventy-one amino acid metabolism-related genes were differentially expressed under conditions of salt stress (Figure 8). Among them, 34 genes were upregulated, including prolyl 4-hydroxylase, probable phospholipid hydroperoxide glutathione peroxidase, probable glutathione S-transferase, probable polyamine oxidase, adenosylhomocysteinase, alanine aminotransferase 2-like, and serine acetyltransferase 1, chloroplastic-like genes. In particular, the FPKM value of a serine acetyltransferase 1, chloroplastic-like gene (evm.TU.CHR17.153) was upregulated by 6.68-fold after 24 h of stress (Figure 8, Table S11). Twenty-six genes were downregulated, including glycine dehydrogenase, probable prolyl 4-hydroxylase 4, aminotransferase, methionine gamma-lyase-like, and serine hydroxymethyltransferase precursor. The FPKM value of an aminotransferase (evm.TU.CHR141065) was 1060.96 at 0 h, which was decreased by 81.98% after 24 h of salt stress (Figure 8, Table S11).

#### 2.6.3. Expression of DEGs Related to Ion Regulation Induced by Salt Stress

**Figure 9:**
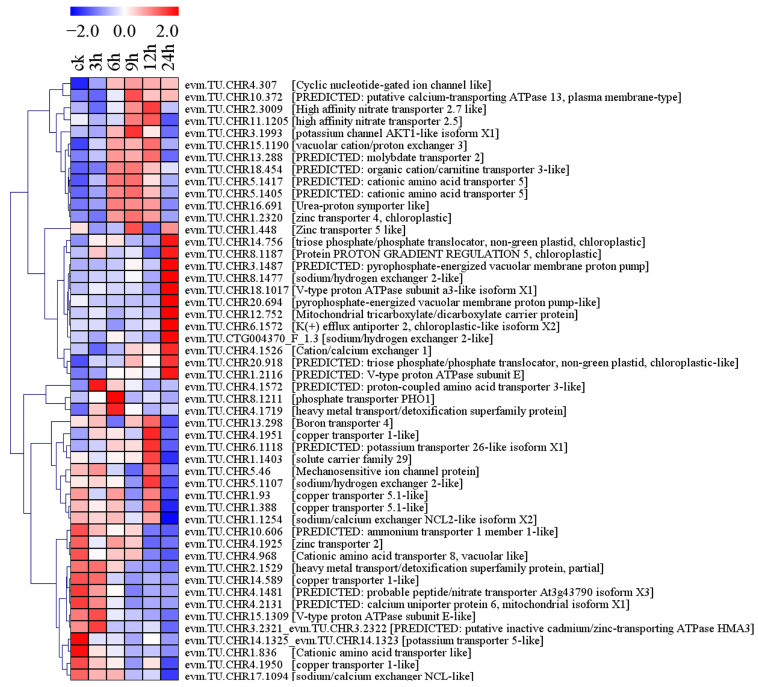
Ion regulation-related genes were differentially expressed in response to salt stress in holly.

Fifty genes related to ion regulation were differentially expressed under conditions of salt stress, including K(+) efflux antiporter 2, V-type proton ATPase subunit, putative calcium-transporting ATPase 13, potassium channel AKT1-like, potassium transporter, pyrophosphate-energized vacuolar membrane proton pump-like, sodium/calcium exchanger, sodium/hydrogen exchanger 2-like, cation/calcium exchanger 1, calcium uniporter protein 6, vacuolar cation/proton exchanger 3, and heavy metal transport/detoxification superfamily protein (Figure 9, Table S12). Among them, two of the three V-type proton ATPase subunit genes (evm.TU.CHR18.1017 and evm.TU.CHR1.2116) were upregulated by salt stress for 24 h, while the expression level of one (evm.TU.CHR15.1309) showed a decreasing trend with prolongation of salt stress (Figure 9, Table S12). Under normal conditions, the FPKM value of the putative calcium-transporting ATPase 13 gene (evm.TU.CHR10.372) was <1, but >1 after 6, 9, 12, and 24 h of salt stress (Table S12). The expression levels of a potassium channel AKT1-like gene (evm.TU.CHR3.1993) and a potassium transporter 26-like gene (evm.TU.CHR6.1118) both initially increased and then decreased with salt stress time (Figure 9, Table S12). Sodium/calcium exchange NCL2-like gene (evm.TU.CHR1.1254) and NCL-like gene (evm.TU.CHR17.1094) were significantly downregulated after 24 h of salt stress. In contrast, two sodium/hydrogen exchanger 2-like genes (evm.TU.CHR8.1477 and evm.TU.CTG004370_F_1.3) were significantly upregulated by salt stress for 24 h. The FPKM value of cation/calcium exchange 1 (evm.TU.CHR4.1526) was <1 under normal conditions but >1 under conditions of salt stress for 9, 12, and 24 h (Figure 9, Table S12).

### 2.7. Changes in ROS Content and Expression of Antioxidant-Related DEGs in Holly under Conditions of Salt Stress

**Figure 10:**
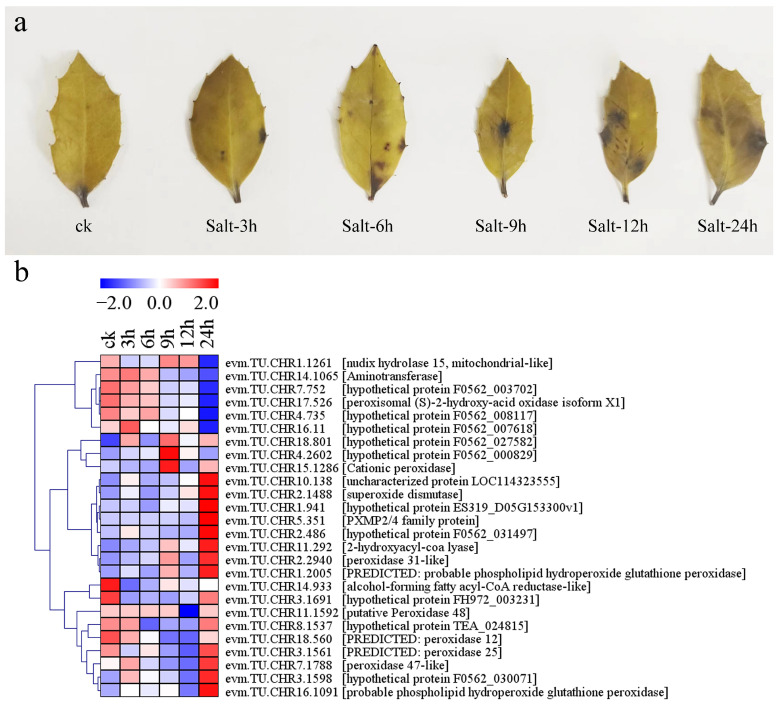
Changes in reactive oxygen species content and expression of antioxidant-related differential genes in leaves of holly under salt stress. (**a**) Results of NBT staining on leaves. (**b**) Antioxidant-related genes were differentially expressed in response to salt stress in holly.

Under normal conditions, there were almost no spots of staining with nitro blue tetrazolium (NBT) on the leaves of holly. At 3 h of salt stress, obvious spots appeared on the leaves, and the staining area on the leaves gradually increased with prolongation of salt stress, indicating the accumulation of ROS (Figure 10a). Plants generally clear ROS through antioxidant mechanisms to reduce oxidative damage. A total of 26 genes related to antioxidants and antioxidant enzymes were differentially expressed under conditions of salt stress, consisting of 18 peroxisome-related genes and 8 peroxidase-related genes. The peroxisome-related genes included four EPHX2 genes, three HAO genes, two MPV17 genes, two SOD genes, one PRDX5 gene, one AGT gene, one FAR gene, one HPCL2 gene, one NUD12 gene, one NUD7 gene, and one PIPOX gene (Figure 10b, Table S13). Interestingly, the two EPHX2s (evm.TU.CHR10.138 and evm.TU.CHR7.752) showed the opposite changes in expression, with the expression of evm.TU.CHR10.138 increasing slightly at 3, 6, 9, and 12 h of stress and then more than doubling at 24 h, while that of evm.TU.CHR7.752 decreased slightly at 3 h, 6 h, 9 h, and 12 h of stress, and then decreased by more than 50% at 24 h. The transcript levels of two HAO genes (evm.TU.CHR17.526 and evm.TU.CHR4.735) showed decreasing trends with an increasing period of salt stress. In particular, evm.TU.CHR17.526 is annotated as peroxisomal (S)-2-hydroxy-acid oxidase isoform X1, and its FPKM value was 1893.16 under normal conditions, which decreased to 437.68 at 24 h of salt stress. Two MPV7 genes (evm.TU.CHR5.351 and evm.TU.CHR18.801) were upregulated under salt stress for 24 h, with the expression of evm.TU.CHR5.351 increasing by 8.84-fold. Similarly, the transcript levels of two SOD genes (evm.TU.CHR2.486 and evm.TU.CHR2.1488) were also upregulated by 24 h of salt stress. evm.TU.CHR2.1488 showed almost no expression under normal conditions, but the FPKM values were 1.17 and 2.32 after 12 and 24 h of salt stress, respectively. The expression level of evm.TU.CHR2.486 was upregulated by 2.57-fold by 24 h of salt stress. Among the eight peroxidases, five genes (evm.TU.CHR15.1286, evm.TU.CHR2.2940, evm.TU.CHR7.1788, evm.TU.CHR1.2005, and evm.TU.CHR16.1091) were upregulated to varying degrees by 24 h of salt stress. In contrast, evm.TU.CHR18.560, annotated as a possible peroxidase 12, was downregulated to varying degrees by various periods of salt stress (Figure 10b, Table S13).

### 2.8. Differential Expression of Salt Stress-Responsive Protein Genes

**Figure 11:**
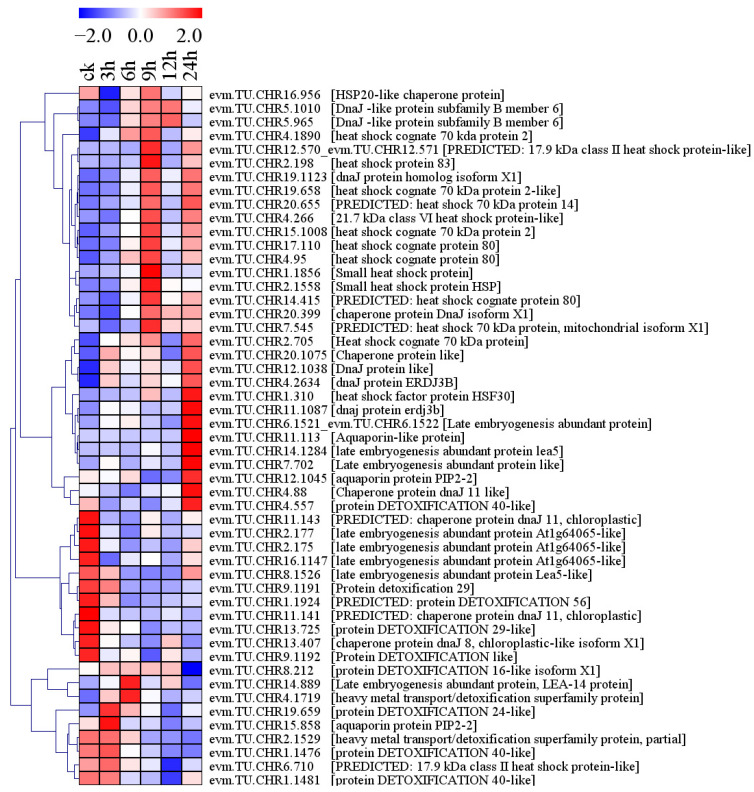
Differential expression of salt-responsive protein-related genes in holly.

Fifty-one salt stress-responsive proteins were found in holly, including 29 heat shock proteins (HSPs)/dnaJ proteins, 3 aquaporin proteins, 8 late embryogenesis abundant (LEA) proteins, and 11 detoxification proteins (Figure 11). Nineteen HSPs/dnaJ proteins were upregulated. Among them, the level of evm.TU.CHR2.705 expression was upregulated by 7.98-, 9.36-, 13.01-, 2.66-, and 14.81-fold after 3, 6, 9, 12, and 24 h of salt stress, respectively. Two small HSPs (evm.TU.CHR1.1856 and evm.TU.CHR2.1558) were significantly upregulated by salt stress. Three dnaJ proteins were downregulated by salt stress. Among them, evm.TU.CHR11.143 had a FPKM value of 2.70 under normal conditions but showed almost no expression under conditions of salt stress. Under normal conditions, the FPKM value of evm.TU.CHR11.141 was 15.65, and it showed almost no expression at 6, 9, and 24 h under salt stress. Two of the eight LEA proteins (evm.TU.CHR14.1284 and evm.TU.CHR6.1521_evm.TU.CHR6.1522) were upregulated, while three LEA proteins (evm.TU.CHR16.1147, evm.TU.CHR2.177, and evm.TU.CHR8.1526) were downregulated by salt stress. Two of the 11 detoxification proteins (evm.TU.CHR19.659 and evm.TU.CHR4.1719) were upregulated by salt stress, while 5 detoxification proteins (evm.TU.CHR13.725, evm.TU.CHR9.1192, evm.TU.CHR1.1481, evm.TU.CHR9.1191, and evm.TU.CHR1.1924) were downregulated. Among them, evm.TU.CHR9.1191 had a FPKM value of 2.15 under normal conditions, but it began to decrease after 3 h of salt stress, and it showed almost no expression at 6, 9, 12, and 24 h (Figure 11, Table S14).

### 2.9. Validation of RNA-seq Data Using qRT-PCR

**Figure 12:**
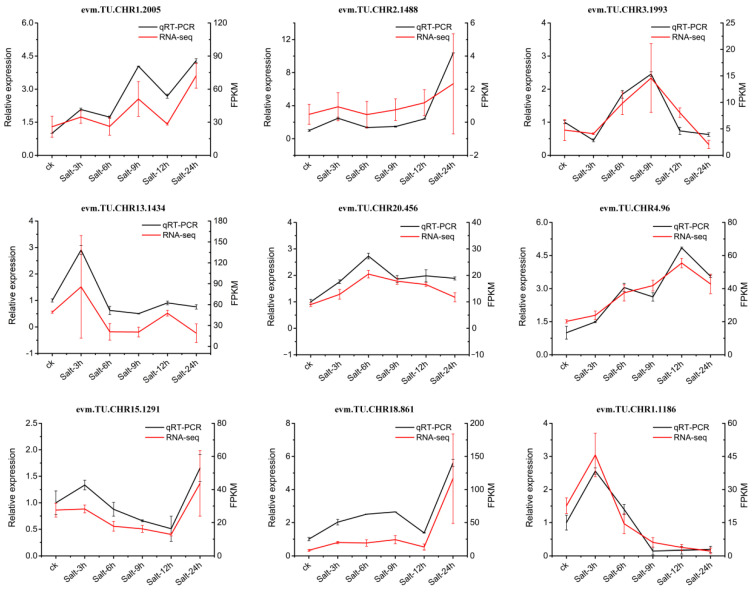
Quantitative real-time PCR analysis of nine DEGs from holly. The relative transcript levels of the nine genes were measured by qRT-PCR, whereas their FPKM values were determined via RNA-seq.

To validate our RNA-seq findings, nine genes (evm.TU.CHR2.1488, evm.TU.CHR1.2005, evm.TU.CHR3.1993, evm.TU.CHR13.1434, evm.TU.CHR20.456, evm.TU.CHR4.96, evm.TU.CHR15.1291, evm.TU.CHR18.861, and evm.TU.CHR1.1186) were selected for qRT-PCR. The primers utilized in this analysis are detailed in Table S15. The trends of changes in transcript levels determined by qRT-PCR were basically consistent with the results of RNA-seq analysis, indicating that the RNA-seq data obtained in this study were reliable (Figure 12).

## 3. Discussion

### 3.1. Ion Homeostasis and Osmotic Regulation in Holly under Conditions of Salt Stress

Ca^2+^ signaling pathways have been reported to mediate plant responses to salt stress, and Ca^2+^ transporters play roles in abiotic stress and development-triggered Ca^2+^ signaling pathways [22]. In holly, the calcium-transporting ATPase gene (evm.TU.CHR10.372), which serves as a Ca^2+^ transport protein, was upregulated by salt stress (Figure 9). SOS3 is the first cloned SOS gene, which can decode Ca^2+^ signals induced by salt (NaCl) stress [23,24]. The most similar functionally characterized protein to SOS3 is the calcineurin B-like protein (CBL) [25]. In holly, five calcium-binding proteins (evm.TU.CHR9.894, evm.TU.CHR14.1028, evm.TU.CHR13.1373, evm.TU.CHR5.1685, and evm.TU.CHR14.947) and one CBL (evm.TU.CHR4.96) were upregulated by salt stress (Figure 4). SOS3 interacts with SOS2/CIPK24 and activates the formation of Ca^2+^ sensor-kinase complexes [26,27]. Subsequently, it phosphorylates and activates SOS1, which plays a role in Na^+^ extrusion and long-distance Na^+^ transport in plants [28]. One CIPK gene (evm.TU.CHR10.386) in holly was upregulated by salt treatment for 3, 6, 9, and 24 h, with an 8.92-fold upregulation at 24 h compared to 0 h (Figure 5, Table S7). Meanwhile, two Na^+^/H^+^ exchange 2-like genes (evm.TU.CHR8.1477 and evm.TU.CTG004370-F_1.3) showed almost no expression under normal conditions and were significantly upregulated after 24 h of salt stress (Figure 9, Table S12). Vacuolar sequestration of Na^+^ is mediated by a vacuolar membrane-oriented Na^+^/H^+^ antiporter in a manner dependent on the proton gradient generated by the vacuolar membrane proton ATPase and vacuolar pyrophosphatase [29,30]. The salt stress led to an increase in the transcript levels of four holly genes (evm.TU.CHR18.1017, evm.TU.CHR1.2116, evm.TU.CHR20.694, and evm.TU.CHR3.1487). These genes were identified as isoform X1 of V-type proton ATPase subunit a3-like, V-type proton ATPase subunit E, pyrophosphate-energized pump-like of vacuolar membrane proton, and pyrophosphate-energized pump of vacuolar membrane proton, respectively (Figure 9). These results indicated that genes related to calcium transporting ATPase, CML, CBL, and CIPKs, Na^+^/H^+^ exchange, V-type proton ATPase, and pyrophosphate-energized vacuolar membrane proton pump regulate ion homeostasis under conditions of salt stress in holly through the Ca^2+^ signaling pathway.

As a way for plants to adapt to their surroundings, salt triggers the production and accumulation of compatible osmotic agents, which serve to lower cellular osmotic potential and stabilize both protein and cell structures [31,32]. Galactitol is involved in tolerance to drought, high salinity, and cold stress. As an osmotic regulator, galactitol is accumulated by galactitol synthase (GolS) and hydrolyzed by alpha-galactosidase (AGAL) [33,34]. In holly, two GolS-related genes (evm.TU.CHR11.1189 and evm.TU.CHR11.1188) were upregulated by salt stress, while two AGAL-related genes (evm.TU.CHR3.1181 and evm.TU.CHR11.1614) were downregulated (Table S10). It has been reported that GolS is also a key enzyme for the synthesis of raffinose, which is used for the transportation and storage of carbohydrates and as a compatible solute to resist abiotic and biological stresses [35]. These observations suggest that the tolerance of holly may be increased through the accumulation of galactitol and raffinose under conditions of salt stress. In addition, the accumulation of proline is also a very important measure to alleviate high-salinity stress [36,37,38]. Ferredoxin-dependent glutamate synthase catalyzes an important step in the glutamate biosynthesis pathway [39]. Glutamate, the primary precursor of proline [40], can contribute to the synthesis of proline by inducing ferredoxin-dependent glutamate synthase under salt stress. For example, the activity of ferredoxin-dependent glutamate synthase was shown to increase significantly under conditions of salt stress [39]. Similarly, the expression of evm.TU.CHR19.183 (glutamate synthase 1) in holly was increased by salt stress, which may lead to the accumulation of proline in holly (Table S10). Many of these compatible solutes are nitrogen-containing compounds, such as proline [41]. Ammonium transporter protein (AMT3-2) and nitrate transporter protein (NRT) play important roles in nitrogen transport and critical roles in ion homeostasis [41,42]. Two high-affinity nitrate transporter genes (evm.TU.CHR11.1205 and evm.TU.CHR2.3009) were significantly increased by salt stress for 9 h and 12 h, but the ammonium transporter 1 member 1-like (evm.TU.CHR10.606) was downregulated by salt stress (Figure 9, Table S12). These observations indicated that nitrate transporters and ammonium transporters in holly may play positive and negative regulatory roles, respectively, under conditions of salt stress.

In summary, the Ca^2+^ signaling pathway and some genes related to the biosynthesis, metabolism, and transportation of osmotic agents in holly may regulate the osmotic and ion homeostasis of cells under conditions of salt stress. This would enable holly to regain stable physiological balance and adapt to salinity stress.

### 3.2. ABA-Mediated Salt Stress Response in Holly

ABA is a well-known internal signal enabling plants to withstand adverse environmental conditions [43]. The levels of ABA are controlled by the balance between its biosynthesis and catabolism. Salt stress activates ABA biosynthesis-related genes in a calcium-dependent manner, resulting in the accumulation of ABA. The primary regulatory step of ABA biosynthesis in higher plants is the oxidative cleavage of *cis*-epoxide carotenoids, a process catalyzed by 9-*cis*-epoxycarotenoid dioxygenase (NCED). Salt stress strongly induces the expression of SgNCED1 [44]. *Arabidopsis* CYP707As is the key enzyme involved in ABA oxidative metabolism. Salt stress induces an increase in levels of the CYP707A transcript, with an especially strong induction of CYP707A1 and CYP707A4 expression [45]. Similarly, the transcript levels of two holly genes (evm.TU.CHR18.868 and evm.TU.CHR4.738) were significantly increased by salt stress, and these two genes were annotated as 9-*cis*-epoxycarotenoid dioxygenase 3-1 and abscisic acid 8′-hydroxylase 4, respectively (Table S1). evm.TU.CHR4.738 showed almost no expression under normal conditions, but its expression gradually increased with the prolongation of salt stress treatment time, with a FPKM value of 18.82 at 24 h of salt treatment. The ABA signaling pathway’s dual negative regulatory mechanism consists of the ABA receptor (PYR/PYL/RCAR), PP2CA, and sucrose nonfermenting 1-related protein kinase 2 (SnRK2), alongside transcription factors like ABRE binding factor ABF and their subsequent targets [46]. Under conditions of salt stress, barley PP2CA and ABC transporters were upregulated, while SnRK2s and PYR/PYL/RCARs were downregulated [47]. Levels of CoABF3 and CoABF7 show a positive correlation with ABA concentration and are notably increased due to salt-induced stress [48]. Similarly, in holly, almost all PP2CA and ABF-related DEGs were upregulated, and five of eight PYR/PYL-related genes were downregulated by salt stress. However, two SnRK2-related genes were upregulated by salt stress (Figure 6). There have also been reports that salt treatment activates SnRK2 kinase activity [49], and salt stress-mediated SnRK2 activation is independent of ABA signaling [50,51]. The biosynthesis, metabolism, and signal transduction of ABA play important roles in the adaptation of holly to salt stress. However, non-ABA-dependent pathways may also be involved.

### 3.3. Antioxidant Enzymes and Nonenzymatic Antioxidant Compounds Mediate ROS Clearance in Holly under Conditions of Salt Stress

Salt stress can induce the accumulation of ROS and eventually lead to membrane lipid peroxidation [52,53]. NBT staining demonstrated ROS accumulation in holly with a prolongation of salt stress time (Figure 10a). Salt tolerance shows a positive correlation with the activity of antioxidant enzymes, including SOD, CAT, glutathione peroxidase (GPX), APX, and glutathione reductase (GR). Additionally, the accumulation of nonenzymatic antioxidant compounds, which are crucial for eliminating ROS induced by salt stress, also contributes to enhanced salt tolerance [54,55]. In holly, two SOD genes (evm.TU.CHR2.1488 and evm.TU.CHR2.486) and five peroxidases (evm.TU.CHR15.1286, evm.TU.CHR2.2940, evm.TU.CHR7.1788, evm.TU.CHR1.2005, and evm.TU.CHR16.1091) were upregulated to varying degrees after 24 h of salt stress. Among them, evm.TU.CHR1.2005 and evm.TU.CHR16.1091 are annotated as probable phospholipid hydroperoxide glutathione peroxidase (Figure 10b, Table S13). In addition, some glutathione transferases (GSTs) were differentially expressed under conditions of salt stress. evm.TU.CHR17.528, evm.TU.CHR15.1104, and evm.TU.CHR10.631 were significantly upregulated by salt stress for 9 h. Among them, evm.TU.CHR10.631 is annotated as tau class GSTU36 (Table S11). GSTs have been reported to play roles in detoxification due to their glutathione (GSH) binding activity and affect the redox state of GSH and ascorbic acid. Members of the plant-specific tau GSTs have GPX activity [56]. Ascorbic acid is also an important antioxidant due to its strong reducing ability. l-Galactose-1-phosphate phosphatase and l-gulonolactone oxidase are key enzymes for the biosynthesis of ascorbic acid [57,58]. In this study, evm.TU.CHR1.1549 and evm.TU.CHR16.550 were annotated as L-galactose-1-phosphate phosphatase and L-gulonolactone oxidase 3, respectively, which were upregulated to varying degrees by some salt treatments (Table S10). These observations indicated that ascorbic acid biosynthesis is increased in holly under conditions of salt stress.

In addition, the potential antioxidant properties of carotenoids and flavonoids may effectively increase plant salt tolerance [59,60]. Carotenoids are a class of highly valuable natural pigments and include lycopene, astaxanthin, β-carotene, and lutein [55]. In holly, carotenoid biosynthesis and flavonoid biosynthesis are enriched by salt-induced DEGs at different stages, especially at 24 h (Figure 3, Table S5). A gene involved in the carotenoid biosynthesis pathway, evm.TU.CHR7.1006, annotated as squalene/phytoene synthase (PSY), was upregulated by salt stress for 3, 6, 9, 12, and 24 h (Table S1). PSY has been reported as the key enzyme for the biosynthesis of C40 carotenoid octahydrol lycopene [61]. Similarly, the three flavanone 3-hydroxylase (F3H) genes (evm.TU.CHR18.1059, evm.TU.CHR2.94, and evm.TU.CHR11.925) involved in flavonoid biosynthesis were upregulated by salt stress for 3, 6, 9, and 24 h (Table S1). F3H is responsible for the biosynthesis of flavonoids [61].

In general, holly may accumulate antioxidant enzymes (SOD, GPX) and nonenzymatic antioxidant compounds (carotenoids, flavonoids, and ascorbic acid) to eliminate ROS and enhance salt tolerance under conditions of salt stress.

### 3.4. Regulation of Transcription Factors in Holly under Conditions of Salt Stress

TFs play important roles in the mechanisms underlying responses and tolerance to various abiotic stressors [62]. They are not just regulators of gene expression but also endpoints of numerous signaling pathways [14]. The expression levels of various TF family genes in different plants are affected by salt stress. For example, genes in 39 TF families in cotton roots were shown to be differentially expressed under conditions of salt stress, and the top-ranking TF families were AP2-EREBP, WRKY, NAC, MYB, and C2H2 [63]. The genes in the 13 TF families in potato were reported to be differentially expressed under conditions of salt stress, including AP2/ERF, ERF, bHLH, ZIP, WRKY, etc. [14]. In this study, when holly was exposed to salt stress, a total of 391 genes belonging to 39 TF families showed changes in expression. According to the number of DEGs, from most to least, they were ERF, bHLH, MYB, MYB-related, NAC, etc. (Figure 7). This indicated that the TF families affected by salt stress are relatively conserved among plants, but the degree of impact varies depending on species. These TFs have been reported to play important roles in the salinity stress response. For example, overexpression of SERF1 in rice significantly improved plant salt tolerance, whereas SERF1 deficiency led to salt sensitivity. SERF1 may generate salt tolerance by amplifying the MAPK cascade signal activated by ROS and converting the salt-induced signal into an appropriate expression response [9]. Some NAC transcription factors in sorghum and wheat are induced by salt stress [64,65]. Overexpression of NAC TFs can also improve salt tolerance in many plants, such as rice, *Arabidopsis*, and chickpea [66,67]. MYB TFs, such as AtMYB2, AtMYB44, AtMYB41, and OsMYB6, are transcriptionally regulated under conditions of salt stress, which can improve plant salt tolerance [68,69]. MYB-associated RADIALIS-LIKE3 (OsRL3) in Oryza sativa facilitates leaf aging and postpones the reaction to salt stress via the ABA signal transduction pathway [70]. Several bHLH TFs, such as TabHLH39, NtbHLH123, and ZmbHLH55, were found to respond to salt stress in wheat, tobacco, and corn [71,72]. GmWRKY12, ZmWRKY33, GhWRKY34, and TaWRKY75-A have also been reported to play roles in salt resistance [73,74]. However, overexpression of ZmWRKY17 in *Arabidopsis* increases its sensitivity to salt stress, and its expression is upregulated by salt treatment [75]. Under conditions of salt stress, bZIP genes show upregulated expression in salt-sensitive wheat varieties, but the expression is decreased in salt-tolerant varieties [76]. 

In this study, ERF accounted for the largest proportion of differentially expressed TFs. Approximately half (21/40) of the differentially expressed ERF genes in holly were upregulated by salt stress. Among them, three ERFs (evm.TU.CHR17.859, evm.TU.CHR16.1194, and evm.TU.CHR4.443) showed almost no expression under normal conditions and were expressed only under conditions of salt stress. In addition, 13 NACs, 18 MYBs, 10 MYB-related, 13 bHLH, and 7 WRKY genes were upregulated to varying degrees in holly under conditions of salt stress. In addition, 6 NAC, 7 MYB, 8 MYB-related, 10 bHLH, and 8 WRKY genes were downregulated to varying degrees under conditions of salt stress (Table S9). These TFs play crucial roles in responding to salt stress and may positively or negatively regulate the salt stress response of holly. Further studies are required to determine the relations between these TFs and upstream and downstream genes involved in the salt stress response of holly.

## 4. Materials and Methods

### 4.1. Salt Stress Treatment and NBT Staining

*I*. *dabieshanensis* was named by Kan Yao and Mao-bin Deng and acquired from the Nanjing Botanical Garden, Mem. Sun Yat-sen (118°49′55″ E, 32°3′32″ N), Nanjing, China. Twenty cutting *I*. *dabieshanensis* seedlings were taken from the nursery and placed in an artificial climate box at 22 °C, with light:dark = 16 h:8 h. After 14 days of adaptation, 15 of them were taken for salt treatment. A 200 mM NaCl solution was used to irrigate 1/3 of the small flower pot. After self-watering, the third and fourth mature leaves were taken from top to bottom at 3, 6, 9, 12, and 24 h. The control is the third and fourth mature leaves from top to bottom of the cuttings without salt treatment. Immediately after sampling, 0.5 mg/mL of NBT was used for staining. The staining solution was vacuumed into the leaf tissue, and staining was started for 24 h when the leaves sank to the bottom of the bottle (the process needs to be dark). After dyeing, the dye solution was discarded in the conical flask, and a proper amount of fixative (anhydrous ethanol:lactic acid:glycerol was added and mixed evenly at a volume ratio of 3:1:1), and placed in a boiling bath until the chlorophyll completely disappeared. After cooling, anhydrous ethanol was added and rinsed repeatedly 2–3 times to observe the staining of the leaves.

### 4.2. Plant Materials for Transcriptome Sequencing

The study utilized annual cuttings of *I. dabieshanensis* seedlings, each comprising approximately 10 leaves, known for their relatively uniform growth. A 200 mM NaCl solution was employed for the treatment. Samples were collected at six different time points: 0, 3, 6, 9, 12, and 24 h, with six leaves pooled together to form a single sample at each interval. Three biological replicates were established for the experiment. Immediately following collection, the samples were snap-frozen in liquid nitrogen and then stored at −80 °C for subsequent transcriptome sequencing.

### 4.3. Transcriptome Sequencing and Mapping, Screening, and Enrichment Analysis of DEGs

The methods of total RNA extraction, mRNA library construction, and mRNA sequence data processing have been used in our previously published studies [77]. The datasets are available in the NCBI database (https://www.ncbi.nlm.nih.gov/bioproject/PRJNA1066056 accessed on 8 June 2024). Using edgeR3.6.3 software to analyze DEGs, different salt treatment groups were compared in pairs to obtain DEGs. The screening criteria for DEGs were fold change (FC) and false discovery rate (FDR), with a screening threshold of FDR ≤ 0.05 and FC ≥ 2. The GO and KEGG enrichment analysis methods for DEGs have been used in our previously published studies [77].

### 4.4. Construction of a Clustering Heatmap and qRT-PCR Validation

Hierarchical clustering (HCL) was used to construct a clustering heatmap using MeV4.9. The Pearson correlation was set as a distance metric, and the FPKM value was normalized. To verify the RNA-seq results, nine DEGs were analyzed by qRT-PCR. The qRT-PCR protocol used here is described in our previous study [77]. *Actin2* was utilized as an endogenous control gene to measure the relative expression levels using the 2^−ΔΔCT^ method [78]. Data were obtained from three biological replicates and three technical replicates. The results are presented as the average ± standard error of three measurements.

## 5. Conclusions

Our research results indicate that salt stress treatment can lead to changes in the gene expression profile of holly. But the response varies depending on the duration of the stress. The transcriptional analysis and identification results of DEGs indicate that ABA accumulation and signal transduction, ion homeostasis regulation, osmotic metabolism, antioxidant accumulation, and transcription factor regulation may play important roles in the response of holly to salinity stress. This provides valuable information for the study of salt tolerance mechanisms in holly.

## Data Availability

The datasets used in this study are available in the NCBI repository (https://www.ncbi.nlm.nih.gov/bioproject/PRJNA1066056 accessed on 8 June 2024).

## Supplementary Materials

The following supporting information can be downloaded at: https://www.mdpi.com/article/10.3390/plants13121638/s1, Figure S1: Phenotypes of *Ilex* plants under different NaCl concentration (0, 100, and 200mM) treatments. Table S1: All DEGs FPKM values. Table S2: Overlapping analysis of GO terms involving biological processes. Table S3: Overlapping analysis of GO terms involving molecular function. Table S4: Overlapping analysis of GO terms involving cellular component. Table S5: Overlapping analysis of the top 30 KEGG terms. Table S6: FPKM values of differentially expressed Ca signaling pathway-related genes. Table S7: FPKM values of differentially expressed PKs. Table S8: FPKM values of differentially expressed plant hormone signaling pathway-related genes. Table S9: FPKM values of differentially expressed TFs. Table S10: FPKM values of differentially expressed carbohydrate metabolism-related genes. Table S11: FPKM values of differentially expressed amino acid metabolism-related genes. Table S12: FPKM values of differentially expressed ion regulation-related genes. Table S13: FPKM values of differentially expressed antioxidant-related genes. Table S14: FPKM values of differentially expressed salt responsive protein related genes. Table S15: Primers used in qRT-PCR analysis of this study.
