# Supplementary figures and images for: Transcriptome Analysis of the Regulatory Mechanisms of Holly (Ilex dabieshanensis) under Salt Stress Conditions
Source: Plants (Basel). 2024 Jun 13;13(12):1638. doi: 10.3390/plants13121638 (PMC11207398; doi:10.3390/plants13121638)

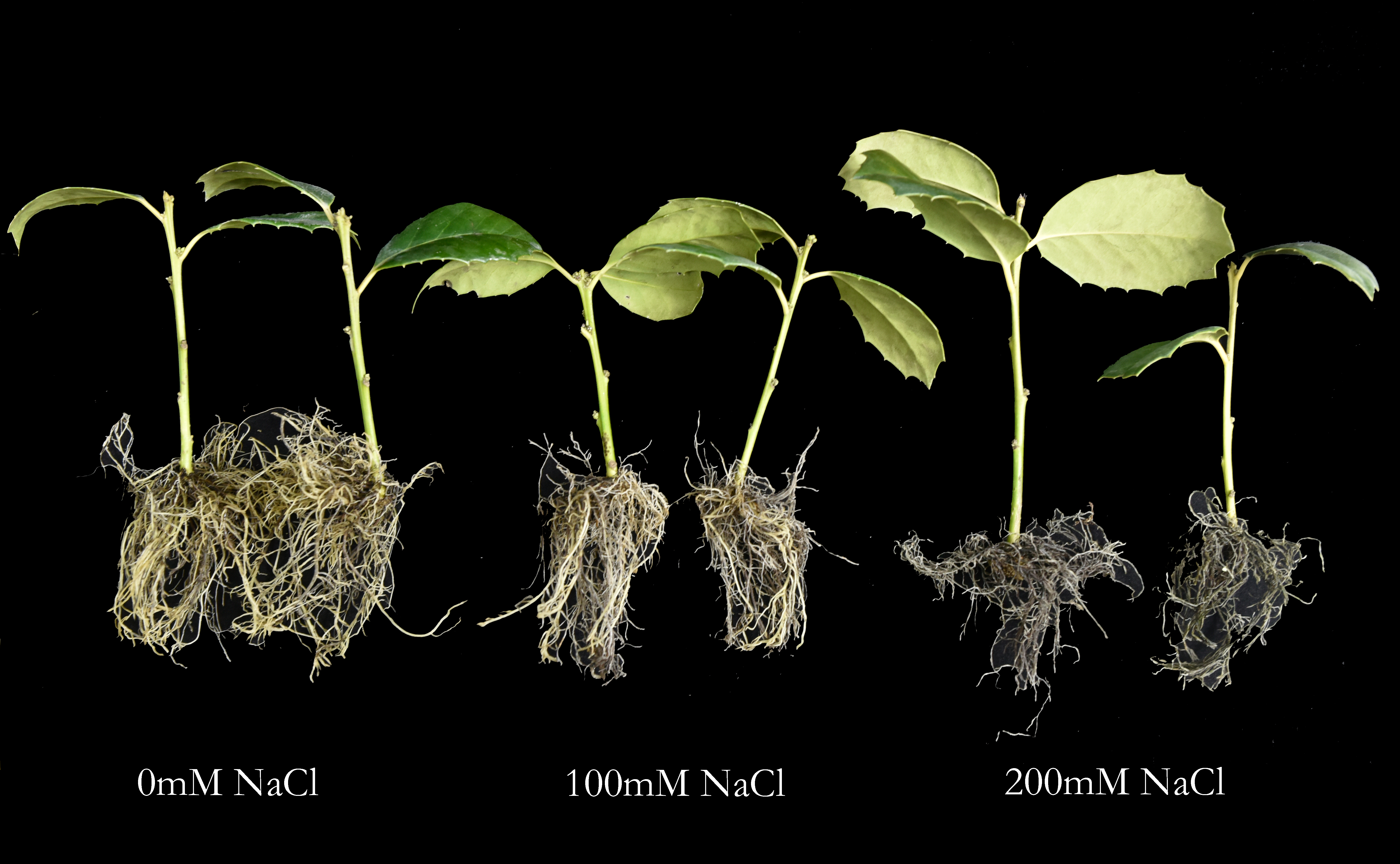

Supplement: Supplementary file 1 [file plants-13-01638-s001.zip › Supplementary material/Supplementary material/Figure S1 Phenotypes of Ilex plants under different NaCl concentration (0, 100, and 200mM) treatments. .jpg]
